# Regional entrepreneurial ecosystems: learning from forest ecosystems

**DOI:** 10.1007/s11187-022-00623-8

**Published:** 2022-04-11

**Authors:** Allan O’Connor, David Audretsch

**Affiliations:** 1 Centre for Enterprise Dynamics in Global Economies, University of South Australia, Adelaide, Australia; 2 Institute for Development Strategies, Indiana University, Bloomington, USA

**Keywords:** Entrepreneurial ecosystem, Regional entrepreneurial ecosystem, Theory building, Socioeconomic, Realist ontology, Analogy, Metaphor, L16, L21, L26, M13

## Abstract

Despite the emerging body of literature on entrepreneurial ecosystems (EEs), theoretical development is still in its infancy. In this article, we explicitly draw upon the analogy of forest ecosystems (FEs) with an EE to extrapolate the regional entrepreneurial ecosystem (REE) as an alternate conceptual framework. The REE considers a region’s socioeconomic activity and the stability of its performance as a whole, influenced by partitioned interests of economics, social arrangements, physical environment, knowledge and the technology that each contributes to the community’s industry and economic order. We contend that it is when an EE is defined by a regional dimension that it is analogous to the study of forests. In this REE analysis, neither the entrepreneur nor their firm are the unit of analysis, but it is the change and stability of the regional socioeconomic ecosystem itself that becomes the priority. Scholars, interested in the effects of entrepreneurship, can learn from ecological studies to more fully grasp the interplay between compositional, structural, and functional elements and specifically how entrepreneurs account for change dynamics.

## Abstract (plain language summary)

When are regional economies like forests? Hardly a day goes by without the term ‘ecosystems’ being bandied about in respect to places, industries and enterprise. Like most terms it is easy to use without stopping to think about exactly what it means or what it implies for our understanding of how our economic world works. This article takes a deep look at how the research of forest ecologies may inform and transfer concepts and approaches to studies of the entrepreneurial ecosystem. We find that by adopting this stance the idea of an entrepreneurial ecosystem, as it is most often presented by academic publications, is turned upside down. In doing so we suggest the purpose, function, and effect of entrepreneurship with respect to how it influences our regional economies comes into focus.

## Introduction

The entrepreneurship literature has generally focused on the analysis of the individual and the firm. However, the emergence of the entrepreneurial ecosystem concept invites a shift toward focusing on regions defined by communities and geographic place. Recently a wave of studies has linked entrepreneurial activity to an ecosystem and context (Acs et al., 2017; Autio et al., 2014; Cohen, 2006; Isenberg, 2010; Malerba & McKelvey, 2018; Mason & Brown, 2014; Spigel, 2017; Shepherd, 2015; Stam, 2015; Szerb et al., 2019) that illustrates the broad adoption of the term ‘entrepreneurial ecosystem’ (EE). A few works have specifically drawn attention to place-based and hence community-based issues (O’Connor et al., 2018; Audretsch & Belitski, 2016), while others have adopted national approaches of the EE concept (Acs et al., 2014). However, questions such as what exactly is meant by an EE and what constitutes a *bona fide* EE, along with a host of conceptual and measurement concerns, are yet to be resolved (Cao & Shi, 2021).

To date, there have been limited efforts to draw correspondence between the EE and ecological studies. Recently, Kuckertz (2019) made an explicit plea for scholars to take the biological metaphor of the EE more seriously. Kuckertz (2019) underscored the importance of valuing multiple stakeholders and introduced the need to take a wider and more subjective account of the EE that ecological framings could potentially reveal. However, the ecological metaphor of an EE has limitations, until or unless the literal extension is applied to express the mechanisms and relationships that underpin correspondence and difference between the metaphorical objects (Tsoukas, 1991).

The field of EE research potentially provides an alternate approach to reconciling an unresolved paradox found in studies of economic geography (Hassink, 2020; Muscio et al., 2015; Oughton et al., 2002). This paradox highlights how greater innovation investment in poorer regions does not simply produce greater innovation outputs because of their relatively low capacity to utilise the investment when compared to the advanced regions. Despite the progress in regional innovation policy, Hassink (2020) argues that to resolve this paradox it requires analysis that moves beyond the economics of a region to take into account the behaviour of political, citizen, and economic actors adopting a socially constructed frame of reference. We feel this provides an opportunity for EE research if we can learn to apply more accurately the principles of ecological studies.

Ecologists have developed approaches to examine the stabilities and instabilities of habitats and populations (Kimmins, 2004). Change, resilience, and whether habitats are threatened or supported in growth or recovery are both important and distinctive areas of work for ecologists. Consequently, ecologists are constantly analysing the balance among demands that in one way or another are pushing and pulling on tipping points that upset the sustainable trajectory of a habitat that supports various interdependent organic species’. If we truly believe entrepreneurship can be applied and developed as a means to grow and develop regional socioeconomic communities, it follows that we must more deeply appreciate how entrepreneurship influences the evolution of regional socioeconomic systems just like ecologists are concerned with how ecosystems sustain, diminish, or expand habitats.

The contribution of our article is a programmatic re-framing of the EE concept, which we distinguish as an REE, or ‘regional entrepreneurial ecosystem’. We achieve this through analogous theorising. Consequently, we question the assumption that entrepreneurship and entrepreneurial activity, whether at individual, firm or industry level, is the exclusive objective priority when applying an ecosystem perspective. We extract, through a more literal extension, an alternate organisation and framework of ideas (Cronin et al., 2021) with respect to the ecological view of entrepreneurship and its application in regional economies. We embrace the suggestion by Pugh et al. (2021) to move beyond our comfort zone and grapple with how a different approach challenges the way the current literature is evolving. We also follow the call to explore more open ideas about the EE and what productive entrepreneurship means with respect to the value it creates (Wurth et al., 2021).

To examine the REE concept more closely and to navigate the challenges of a holistic interdependent ecosystem that is idiosyncratic to a regionally defined community, we establish the forest ecology (FE) model as our theoretical point of departure. This draws upon the popularised metaphorical reference to an EE as a ‘rainforest’, which Hwang and Horowitt (2012) used to explain the mechanisms of an innovation ecosystem such as Silicon Valley. Indeed, Hwang and Horowitt (2012) offer a persuasive argument based on anecdotal evidence and selected science-based studies, woven together into a convincing narrative that promotes the idea that the function of an EE is to beget entrepreneurship. However, we agree with Kuckertz (2019) that the loose metaphorical framing warrants much closer and particular attention. Hence, our guiding research question is: how does the analogy of forest ecosystems inform the study of entrepreneurial ecosystems?

## Analogous theory building

We first and foremost acknowledge the concept of the EE is borrowed from a branch of the biological sciences (Aldrich, 1990) that has a much longer history of dealing with the relations between organisms and their environment than studies of EEs. We seek to inform the rapidly expanding area of EE research, extending its reach into a place-based regional context, and to provide a framework useful for operationalising the interplay between entrepreneurship and ecosystem evolution. Some principles borrowed from the biological field may fit well, while others may not. The resulting conceptual model is propositional in that it appears worthy of empirical testing. However, it is not presented as a statement of fact, rather as an alternate viewpoint to the predominant conceptions of an EE.

The rainforest metaphor (Hwang & Horowitt, 2012) motivated our enquiry. However, in broad categorisation, rainforests do not define all forests, and FEs are concerned with forests of various types forming across a range of climatic conditions. These climatic ‘zones’, in turn, determine the type of vegetation, animal, and microbial communities that can survive and thrive (Kimmins, 2009). Just as forests vary according to climatic and geographic positioning, we can anticipate that EEs will similarly vary in form and structure. However, we do not attempt to construct a typology of this diversity. Rather, we emphasise our primary task is to provide a conceptual arrangement of ideas that enables the study of EEs as relevant to regional evolution.

We follow Tsoukas (1991), whose methodological approach defines both a ‘target’ and ‘source’ domain for an analogous study. The approach is based on ideographic studies grounded in a realist epistemology that specifically examines how real social structures work, exposing causal capabilities regardless of how they may be experienced in an empirical setting (Tsoukas, 1989). In our case the ‘target’ domain is the EE and the ‘source’ or analogous domain is the forest ecosystem (FE). The objective is to map the conceptual relationships between the source domain—which has a longer history and more mature theoretical basis—and the target domain.

We truncate the approach of Tsoukas (1991) for this article, in order to conceptualise an analogous conceptual model of an EE. We first interpret an analogous conceptual model and then examine that model through a realist framing. Our intent is to propose a theoretical re-framing of the EE that portrays new and/or alternate layers of reality to drive new research questions (Tsoukas, 1989). We leverage the FE field, which similarly to an EE identifies with a realist ontology. Just like the field of FE juggles the objective and subjective understandings of a forest to project its sustainability (Kimmins, 2004), drawing the analogy reveals how the objective and subjective understandings of regional socioeconomic activity sustains a community. Autio and Levie (2017) also highlighted the importance of both objective and subjective views for policy-making and management of an EE. Through our analogous theorising, we extend this line of thinking by drawing out the complementarity of the realist and objective framings and elaborating a conceptual model that guides research for this different point of departure. Importantly, we do not seek to fit the FE conceptual model to the EE but draw out the conceptual parallels to extend our understanding of the EE and propose a reconceptualised REE model that offers potential for further testing, true to the realist framing.

In this research we adopt an evaluative approach but stop short of the systems theorising outlined by Tsoukas (1991). Criticism of systems theory development (including complex systems) contends that it commonly creates homologies from unrelated systems in the pursuit of achieving generalisations between them. The accusation is that homologies direct isomorphic theorising to the extent that the homology becomes the governing theory to which an analysis of other systems should fit (Kozlowski & Klein, 2000). Tsoukas (1991) deals explicitly with the critique of isomorphic objectives by arguing that the ultimate goal is to identify a scientific model that would be both valid and useful as a theory to explain both the source and target domains. In other words, the scientific model for systems theorists draws closer to explaining ‘all’ systems by at least defining the coherence between the two in question. However, we do not aim for or claim an isomorphic systems theory agenda in our research; as entrepreneurship theorists this is beyond our intent and we’ll leave the work of systems theorising to the systems theorists. At best we seek to express a homomorphic relationship that suggests the extent to which the conceptual models between the domains appear to have similar structures and theoretical elements, even if named or identified differently, as is relevant to the independent and discreet domains of study.

## Moving from an FE metaphor to an analogous REE

### The informing EE literature and insight

The extant EE literature expressly detects and describes the facilitating contexts within which entrepreneurs make their decisions and are analysed. Maroufkhani et al., 2018) go as far as to say that encouraging successful entrepreneurship is ‘the duty of public leaders and policy makers to design and implement a virtuous cycle’ (2018, p. 546). Presumably, this stance is designed to inform governance (Autio & Levie, 2017; Colombo et al., 2019) and/or to inform innovation and strategic approaches for firms designing their own ecosystems (Jacobides et al., 2018), that is, ones that enhance the frequency, effectiveness, efficiency, and value creation of entrepreneurship (Acs et al., 2017). Consequently, there has been a focus on ecosystem management whereby the intent is to manage the services of an EE by steering the establishment and development of innovative, high-growth ventures to allocate resources toward higher productive use (Autio & Levie, 2017). By contrast, Isenberg (2016), one of the authors who popularised the term EE, questions the assumptions of much of this work and argues that an EE cannot be created, controlled, defined by broad geographies and endowed with intentions or that the entrepreneur is central. Within this background, we find a significant theoretical development opportunity that we attempt to address in this article.

The EE research has been framed by analysing specific outputs and outcomes, and dominant among those is an economic purpose. This is understandably so, given that the field of entrepreneurship is plainly connected to economics and business disciplines. The terms of firm strategy and value creation, (Adner & Kapoor, 2010; Adner, 2017), competitive advantage (Jansson et al., 2014), digital affordances (Autio et al. (2018), productive entrepreneurship (Stam, 2015), regional economic growth (Content et al., 2020) and resource allocations (Acs et al., 2014; Autio & Levie, 2017) are expressly concerned with how entrepreneurs and/or firms prosper, survive, grow and shape the economic fortunes of the proponents of economic activity (see also Audretsch & Belitski, 2021). The primary objective appears to be more and/or better entrepreneurship based on the argument of the increased economic value creation that follows.

A number of studies illustrate how the EE framing has tended to objectify the various patterns of new venturing in a given economy. For instance, the models of an EE have prioritised the new value-adding ventures and productive entrepreneurship as the objective of an EE (see Stam, 2015; Wurth et al., 2021) or have incorporated performance measures that consider the intensity and density of entrepreneurial activity (Spigel & Harrison, 2018; Stangler & Bell-Masterson, 2015) or competitiveness (Sitaridis & Kitsios, 2020). At the extreme, an entrepreneurial ecosystem has been defined through the observation of unicorn businesses (Acs et al., 2017); those businesses that reach a $1 billion valuation in under 10 years. Szerb et al. (2019) describe the moderating role of the EE on producing quantity and quality of entrepreneurship and further relate this to the gross value added per worker as a regional performance measure. Complex system theorists seem to share this view, defining an EE as ‘a self-organized, adaptive, and geographically bounded community of complex agents operating at multiple aggregated levels, whose non-linear interactions result in the patterns of activities through which *new ventures form and dissolve* over time’ (Roundy et al., 2018, p. 5, emphasis added). In each of these cases, the objective analysis of the EE is new venturing and/or the value added by new ventures often prioritised in economic terms.

Critically, we can also detect the presence of alternate objective views being expressed in terms of different outcomes such as knowledge (Clarysse et al., 2014; Horváth & Rabetino, 2019), the environment and sustainability (Brock et al., 2001; Cohen, 2006; Dooley & Letiche, 2009; Geels, 2010; Kay et al., 1999) that may place priority on non-economic forms of value. Interestingly, Audretsch et al. (2019) bring to light the very essence of an ecosystem by referring back to the ancient Greek origins of the term, *oikos*, which is a Greek unit of analysis identified as the household. However, households are not uniquely economic, social, technical or even physical or knowledge-based units of analysis, but instead exhibit a structural combination of each of these features.

Our initial review of the EE literature suggests that the extant research may fall into various categories, each with a focused analysis, largely driven by economic purposes but also for objectives related to the social, knowledge, technological or physical environment. While we acknowledge the value of these approaches, FE studies suggest an alternative. From a FE perspective, an objective or partitioned value of a forest is complemented by an intent to ‘describe and provide an explanation for, and an understanding of, the *differences* between forest ecosystems in different places, and *the changes in any one forest over time*’ (Kimmins, 2004, p. 73, emphasis added). Similarly, an alternate EE view could acknowledge that any given place-based EE potentially has its own unique form. The analysis of place-based EEs may therefore emphasise describing and providing explanations for, and an understanding of, the differences between EEs in different places, and the changes in any one EE over time for which entrepreneurship is at least partly responsible. By treating an EE holistically and as place bound, it draws focus to such questions as how entrepreneurship continually emerges and evolves in any particular place; how differences in places influence the manifestation of different forms of entrepreneurship; what effect different entrepreneurship has on different places; and how different places evolve over time as influenced by variance in entrepreneurship, among other factors. Notably, these questions are not concerned with the objective value of any particular output or outcome but rather draw attention to how entrepreneurship is related to the change in socioeconomic arrangements of places.

In formulaic terms, the two approaches to EE studies may be expressed differently. First, the extant approaches to EE research are concerned with how entrepreneurship is associated with delivering a specific form of value delineated from the other forms of value enabled by the elements of the ecosystem. Hence, entrepreneurship is an operator that produces the potential value generated by the interaction of system elements. Studies, therefore, seek to optimise this relationship and discover idealised ecosystem elements and entrepreneurial activity.

Objective Value = ƒ (Ecosystem elements x Entrepreneurial activity)

The research in this endeavour is driven by disciplinary interests in terms of objective value, and the entrepreneurship theorist will seek to understand, design, and theorise the conceptual relationships between the ecosystem elements and the entrepreneurial activity that drives gains in the objective value. While a worthwhile and meaningful exercise, it risks prioritising one objective value at the expense of others delivering, for instance, more economic outcome at the cost of environment or knowledge domains.

By contrast, an alternate approach is concerned with the variation in the holistic ecosystem and how entrepreneurship associates with the change and stability of the ecosystem elements over time.

Δ Ecosystem = ƒ (Ecosystem elements / Entrepreneurial activity)_t1_ - (Ecosystem elements / Entrepreneurial activity)_t0_

Framing research in this way leverages the systems’ perspective, and the focus of the study is on how the system is influenced by shifts and changes among the elements and the entrepreneurial activity. It is not strictly a systems science as the entrepreneurship scholar is less concerned with how these systems contribute to the universal idea of systems theory but instead how the specific systems that support and sustain socioeconomic activity are affected by the entrepreneurial activity. Hence, ecosystems theory differentiates from systems theory because ecosystem theorists are concerned with the ecological relationships between organisms and their environment. Furthermore, the concern of the entrepreneurship theorist in this view is not intending to optimise an objective value but instead explain how the ecosystem is stabilised or destabilised by the entrepreneurial activity.

While neither approach has supremacy over the other, if studies of an ecosystem do not appreciate and engage in both approaches, then it potentially reduces the completeness of EE studies and the contributions that these studies make to regional development.

Relevant to this is the idea proposed in a life-systems context of population ecology (Sharov, 1992, p. 485):

A system is a combination of unity and partition both in space and time: if there is no unity then there is no system, if there is no partition then a system turns into a simple object that can be described externally, but not explained.

The key insight that inspires this research is that the place-based EE itself is a composite structural unit that includes a bundle of economics, technology, social arrangements, institutions, knowledge, physical environment, and entrepreneurship occupying a particular place and time. In concert, each aspect contributes to the basic socioeconomic order of a regionally defined community. From an entrepreneurship scholar’s vantage point, we may not only be interested in the generation of entrepreneurial activity and/or any one or other value objective but moreover, how the entrepreneurial activity emerges and influences (or not) any particular change in a regional community’s socioeconomic order. We perhaps should question how over time entrepreneurship engages with the differing value objectives of social, economic, technological, institutional, knowledge, and physical/natural environment to balance or disrupt the socioeconomic order of a community.

From this standpoint, entrepreneurship, the economy, social arrangements, institutions, knowledge, technology, and physical environments are not individual and divisible determinants of the ecosystem and nor does any single emphasis on one aspect or another illustrate how an EE works. Furthermore, neither does any one aspect indicate the EE’s existence but each are instead attributes of the EE due to its arrangements of and interactions between the elements that portray the structure of the social and institutionalised arrangements of a particular regionally defined community. It is the bundled value attributes of the regional level community that is the portrayal of a place-based ecosystem and it is the study of the effects and influences of entrepreneurship as a change mechanism that we argue distinguishes the study of place-bound EE or an REE.

We contend that it is when an EE is defined by a regional dimension that it is analogous to the study of forests. Forests are a spatial unit which inherently contain partitioned elements and interests among competing species and stakeholders that take different forms, influenced by various geographic and climatic conditions. EEs when taken as a regional unit of analysis are also spatially arranged with partitioned competing interests among its people, communities, institutions, and natural environment that also vary in form across the globe due to different conditions. Just like forests, EEs exhibit variation dependent on geography, location, and institutional contexts akin to climatic zones and topography in forest ecology studies. From this point we distinguish then the general concept of the EE to the specifics of a regionally defined community EE as a regional entrepreneurial ecosystem (REE).

### The conceptual model of a forest

A widely held view in terrestrial ecology, and consistent with the EE of Hwang and Horowitt (2012), is that an ecosystem approach ‘addresses the interactions that link the biotic systems, of which people are an integral part, with the physical systems on which they depend’ (Chapin et al., 2011, p. 3). Furthermore, the stability of this basic concept is evident when definitions stretching back to as early as the mid to late nineteenth century define the ecosystem similarly as ‘the study of the natural environment including the relations of organisms to one another and to their surroundings’ (Haeckel, 1869 in Odum & Barrett, 2005). The forest definition (Kimmins, 2004) stems from this fundamental view and can be stated, in general terms, as a terrestrial ecosystem within which the inter-relational processes between communities of organisms and their environment are supported by a dominant system of trees.

Despite the consistency of ecosystem definition over time, it can be further noted that an FE is commonly observed through differing lenses. Chazdon et al. (2016) describes the various vantage points that alter the definitions of the forest, including:

a source of timber products, an ecosystem composed of trees along with myriad forms of biological diversity, a home for indigenous people, a repository for carbon storage, a source of multiple ecosystem services, and as social-ecological systems, or as all of the above. (Chazdon et al., 2016, p. 539)

In other words, FEs can be studied through various utilitarian lenses that bias the study toward maintaining or managing some particular objective value. By contrast, in the studies of forest ecologies, Kimmins (2008) claims that:

[r]educing complexity to facilitate disciplinary hypothesis testing is a necessary part of ecological sub-disciplines, but for the products of reductionist, disciplinary science to be useful in society’s quest for a sustainable relationship with forests, the pieces of the scientific jigsaw puzzle must be integrated into a complete picture, and the picture projected forward over time to create a ‘movie’ of possible forest futures. (p. 1625)

As a first theoretical point, we find in both forest (Kimmins, 2008) and, not coincidentally, population (Sharov, 1992) ecological studies there are two contrasting points of view, one is disciplinary focused and an objective standpoint of some element of the system and the other is of the holistic ecosystem and its interdependent ordering. The studies of EEs therefore are likely to exhibit similar characteristics whereby there are useful sub-disciplinary interests in objective elements of the system but equally there is a need to focus on regionally defined communities and the projection of the integrated and possible futures of regional place-based socioeconomic activity.

Drawing deeper on the FE metaphor, it is notable that there is a primary concern with temporality and change. For instance, Eldridge et al. (2016) emphasise the change induced by the grazing habits of livestock on ecosystem composition, structure and function. Setälä (2002) examined how reductions in ecosystem complexity influenced by below ground food systems influenced above ground performance in the growth of birch and pine seedlings, for example. Introducing the human element, Chazdon et al. (2016) examined the change effect of policy inferences on forest degradation, deforestation, regrowth, and the restoration of forests in forest management and assessment. Weber and Flannigan (1997) investigated various scenarios of climate change to determine the possible effects that may be imposed on boreal forest ecosystem structure and function. In sum, understanding and explaining change is a primary concern and thereafter, an objective management viewpoint can be applied to influence future change in one prioritised direction or another.

**Fig. 1:**
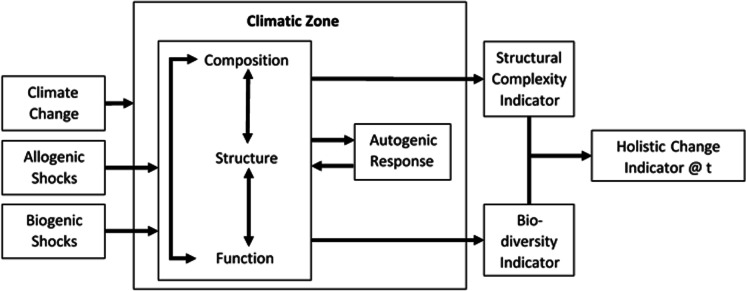
A forest ecosystem conceptual model ( Source: authors)

The forest ecology literature (see for instance Kimmins, 2004; Maleki & Kiviste, 2015; Setälä, 2002; Weber & Flannigan, 1997; McElhinny et al., 2005; Eldridge et al., 2016) can be synthesised to suggest three primary attributes that constitute a forest ecosystem’s configuration: structure, function and composition (see Fig. 1). In a report on the sustainable management of forests, McElhinny (2002, p. 3) states:

- ‘Structure refers to the spatial arrangement of the various components of the ecosystem, such as the heights of different canopy levels and the spacing of trees;
- Function refers to how various ecological processes, such as the production of organic matter, are accomplished and to the rates at which they occur;
- Composition refers to the identity and variety of elements, as characterised by species richness and abundance’.

However, it is apparent that these attributes are not easily or clearly delineated and there is inherent interdependency between them. For example, McElhinny (2002, p. 4) explains how the presence of structural attributes can be indicators of function (e.g. dead wood as a structural attribute provides the conditions for the functional attribute of decomposition and nutrient recycling) or that species composition is elementary to structural attributes (e.g. a forest composition of a species of trees can provide structural attributes such as tree hollows, flowering or shedding of bark).

This interdependency between attributes causes many conflicting views about terminology, measurement, constructs, and methods in the FE literature. For instance, Ehbrecht et al. (2017) explain how uneven-aged, multi-species stands of forest trees may be termed either structural complexity, structural heterogeneity or structural diversity. Furthermore, they offer that there is no widely accepted definition of structural complexity or the related terms and the methods of measurement of structural complexity vary greatly. It seems that this is based in the first place on the fact that there is also no broadly agreed or definitive set of structural attributes (Ehbrecht et al., 2017, McElhinny, 2002). As a result, Ehbrecht et al., 2017) concluded that there remained uncertainty about what ‘the most appropriate measure of stand structural complexity is, since different measures deal with different aspects of complexity’ (p. 8).

While the interdependency of the three primary attributes may produce much of the uncertainty in the specifics of measurement and management, there do appear to be two strong indicators of interest when it comes to the change dynamics and trajectories of forest ecosystems. The first is structural complexity (as noted above) and the second is biodiversity (Chazdon et al., 2016). Notably, rainforests are among the most structurally complex and biodiverse of the forest ecosystems offering species-rich contexts (Swain & Whitmore 1988; Delarue et al., 2015). Biodiversity within a forest is an important indicator of ecosystem resilience or the ability of the ecosystem to bounce back to conditions prior to any climatic change or disturbance (Kimmins, 2009). Meanwhile, the greater the structural complexity of the vertical and horizontal patterns or arrangements of compositional elements, the greater the complexity of interactions between the living organisms, the dead organic matter, and the physical environment (Kimmins, 2009). Zhang et al. (2010) note that a holistic approach is increasingly needed in ecosystem studies, ‘based on properties of the whole system rather than just its components’ (p. 694) suggesting that these two indicators should also not be treated solely independently.

In addressing changes in a FE, four types of change factors (Kimmins, 2009) are identified that occur over differing time scales and can affect the structural complexity and biodiversity of a forest. There are three externally originated change influences and one internally originated (see Fig. 1). The first of the three external change mechanisms is the millennial long-term changes in climate or *climate* change. Next are the relatively more frequent *allogenic* changes that may occur over a century or two, or every few decades or less in some instances, that arise from physical disturbances such as fire, wind, snow, and landslides. The third level of external change is *biogenic,* which stem from insect plagues or biological disease epidemics that can alter structural complexity and impact biodiversity. The fourth is *autogenic* change, which describes altered internal conditions in response to the external disturbance, such as shifts in soil and microclimate, plant, animal and microbial species composition, structure and function. Autogenic change is part of ecological succession in response to the disturbances imposed by the millennial, allogenic and/or biogenic factors that alter rates, patterns and directions of successive states of the forest ecology.

### The analogous REE conceptual model

**Fig. 2:**
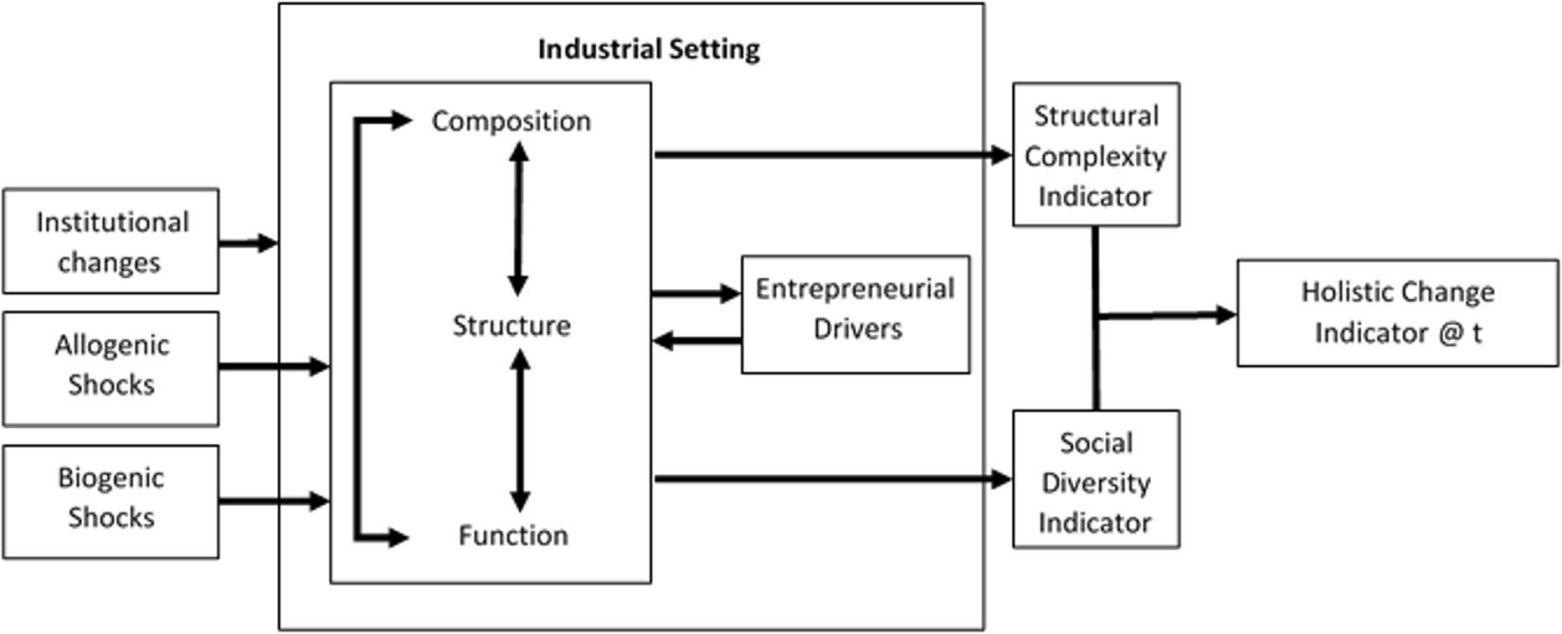
Analogous conceptual model of the EE defined as an REE ( Source: authors)

We now draw upon the FE conceptual framework to construct an analogous model for the REE (see Fig. 2).

The first question is one of definition. If FEs are defined by the various types and forms of trees that range across climatic zones, then, can REEs could be analogously defined by the various forms of entrepreneurship that range across certain globally defined industrial zones? In the same way that different species of trees are supported by different climatic conditions, various forms of entrepreneurship such as is argued through studies by Audretsch and Belitski (2021); Morris et al. (2015); Welter et al. (2017); or Pahnke and Welter (2019) may be supported by different industry conditions. This we might call the industrial setting. and this setting may at least partially influence the presence of entrepreneurs and the entrepreneurial activity they pursue within a dominant industry profile.

Extending this from a rainforest metaphor, where rainforests are among the most highly structurally complex, biodiverse and species rich of the forest ecosystems, then one would expect that one specific form of REE would also exhibit extremely rich industrial conditions with high complexity in industry structure and abundant social diversity. In support of the analogy, this may relate to the regions that exhibit the highest ‘spikes’ of entrepreneurial rich contexts (Brown & Mason, 2017) across the globe.

However, the inference to be drawn from the FE analogy for an REE would be that REE studies would include all types of regional contexts, not just the ‘spiky’ ones, to explain the differences and variations in order among the various industrial settings. This establishes a contrast to the original metaphor, which was concerned with the functioning, protection, and survival of an ecology that exhibited high entrepreneurial activity in contexts like Silicon Valley (Hwang & Horowitt, 2012). To remain consistent with the FE analogy, the REE research should not solely be concerned with the most entrepreneurially rich or abundant contexts. Instead, it invites studies of difference to explain why and how variations among institutional and industry contexts influence and manifest different circumstances for a variety of entrepreneurial forms. This is consistent with the views of EE scholars such as Stam (2015) and Wurth et al. (2021).

A further inference for REE research is that spatial variation should be accounted for in drawing the analogous parallel. Of relevance to this question is the classification of forest ecologies according to climatic zones (Vogt et al., 1995). On a global scale, regions can be distinguished by common industry ‘zones’ through such terms as primary, secondary, tertiary, quaternary or quinary industry (Sheth, 2017). For instance, in rural agricultural areas, the farming venture may dominate (primary industry), while in densely populated cities it may well be professional service ventures (quaternary industry). The density of firms may be dependent on a dominant economic activity that changes the business unit from acreages in farming businesses to the small and medium businesses in the professional services. As a starting point, the fundamental and dominant industry within a region may be thought analogous to the dominant tree species in an FE. Therefore, the spatial concentration of industry may define both the REE form and the range that supports the inter-relational processes within the REE’s boundaries.

The next question to arise from the analogous extraction is how the notion of change in an FE conceptual model applies to the REE. This would suggest that studies of an REE would be concerned with change within the defined industrial conditions. Understanding change is important if a managerial approach seeks to influence change in one direction or another. If change is of primary concern in FE studies, whether it be for specific objective aspects of management or the general stability of a given ecosystem, then we would also consider change for the REE to be the primary focus of analysis. The four forms of change mechanisms in FE could presumably have an equivalence.

An analogy for the first level of change across millennia, i.e. climate change, might be seen in REE terms as the shifting powers in global economic and trade dominance and/or the longwave technology shifts. Both these aspects of slower (millennial)-paced change could give rise to shifts and changes in the socioeconomic landscape of a community.

We can also translate the allogenic shocks literally when we consider the socioeconomic impact of fire, floods, and other natural disasters. Biogenic change may also be witnessed in literal translation through the socioeconomic impacts of pandemics such as the current day COVID-19, SARS in 2003 or the H1N1 flu in 2009.

The last change mechanism in an FE is the autogenic response induced by external shocks and change drivers. Autogenic change has likely the most profound implication for an REE in that the autogenic response in socioeconomic terms would be the entrepreneurial behaviour that re-shapes the structure, composition and functions within a community. This leads to the next question, what might be the structure, composition, and function definitions in the REE context that are subjected to changing conditions?

If we first consider structure as the spatial arrangement of the various components of a FE, then an REE context may require spatial arrangements or layouts of the physical places and facilities that, within a community, support the industrial activity. More specifically, how this arrangement fosters entrepreneurial activity would come into focus. This might include proximities of universities, large organisations, innovation precincts, hubs and incubators and/or accelerator facilities and business, commercial and/or creative districts and transport facilities or other relevant infrastructure.

Just as in the FE context, the interdependency in the configuration between the three primary concepts (structure, function and composition) also becomes quickly apparent. It is quite plain that in order to understand the structural arrangement of a place, it is also necessary to know what the composition of its various identities, actors and physical elements might be. However, while ‘structure’ examines how the elements are arranged and/or combined, ‘composition’ is concerned with not only what is present but how much of each element is in evidence, what kind of variety is apparent and in what proportion. What natural elements exist within the REE range? What population resides there and in what cultural, professional and other diversity? What physical attributes are constructed and what is the range of housing, factory, warehouse, office space etc. contained and/or available in the REE?

Both composition and structure may also influence function and vice versa. Function in this case would examine the processes that sustain the ecosystem and keep it in balance or be influences of change. In the socioeconomic context, this would include questions about the functions that create and sustain employment and export as well as such functions as social interactions, knowledge exchange, transference or transformation. Are there functions of supply of goods and services to support socioeconomic activity and training or human capital development functions or functions that relate to furnishing basic human needs of food, water and shelter?

The next component of the FE conceptual model suggests that the configuration and interdependency of structure, composition and function combine to suggest two different indicators of complexity and diversity. The first, structural complexity, relates to the ability of the structural composition to give rise to rich species diversity. The analogous question is how the structural arrangement of industry or commerce in a region resembles the various habitats in forest terms where biological diversity can reside, find shelter, hunt for food, or thrive in a suitable nutrient rich and fertile soil condition. In an REE sense, the question raised is to what extent the structural arrangement of the region creates the conditions for diverse populations of socioeconomic actors to conduct business, revitalise, learn, and grow. Does it facilitate trade, exchange, and dynamic marketplaces? Can new businesses gain access to low-cost environments in which to start, and do established businesses have their infrastructural needs? The greater the structural complexity the more opportunity and support there is for the second major combinational indicator used in FE studies, that of biodiversity.

In a socioeconomic sense, while structural complexity indicates the range of habitats to host entrepreneurial activity, the biodiversity, which specifically in FE terms means the biological diversity in microbes, flora and fauna, could translate to social diversity for an REE. In an REE context, higher social diversity would indicate diversity in the community level populations: gender, cultures, knowledge, professions and experiences which, theoretically, drive creativity, innovation, economic resilience and stability. This conceptualisation would split the FE indicators of structural complexity and biodiversity between the following: the building blocks of structural complexity, which provide an array of ‘sites’ for industry and economic activity, and the social diversity that constitutes and enacts the ‘activity’ within an ecosystem, maintaining or disrupting stability and responding to external and internal shocks and change influencers.

### The FE and REE conceptual model correspondence checks

To examine the analogous alignment of the REE conceptual model, we leverage the EE literature and logic to conduct five ‘realist’ correspondence checks between the FE and the place defined REE concept.

#### Check 1: Definitional correspondence

In more recent literature, we can observe resonance between the EE and FE management perspectives, whereby an objective function is imposed on the definition of the ecosystem. As discussed earlier, a number of studies base their definition of an EE by focusing directly on entrepreneurial activity and/or the value outcomes it produces (Acs et al., 2017; Roundy et al., 2018; Spigel & Harrison, 2018; Stam, 2015; Stangler & Bell-Masterson, 2015; Szerb et al., 2019). These views approach the definition of an EE as supporting entrepreneurial activity dependent on the context or setting and are consistent with FE studies in terms of an objective ecosystem perspective. In the case of an EE, the entrepreneurial actor and activities are given priority for management or governance within the EE to produce some objective measure of regional performance, usually, but not always, economic.

However, the extant literature seems to pay little attention to the alternate view of the FE literature, where there is no prioritisation of service or claims on the ecosystem but instead a focus on the stability or otherwise of the ecosystem to sustain, recover or evolve. In this regard there is no apparent observable (within the limits of this study) correspondence between the FE and EE where the holistic views of stability, change and succession of places come into focus. From this perspective, the lack of correspondence between the EE and FE concepts suggests an opportunity for reflection on the role of the entrepreneurial activity in a socioeconomic ecosystem for its influence on a region’s stability. Hence, we propose the REE concept is theoretically analogous to that of FEs with respect to the change mechanisms of a holistic ecology but differs to the extant models of the EE dominant in the literature focused on producing entrepreneurial activity and primarily economic or other objective outcomes.

This suggests a definitional stance on an REE (an alternative to the predominant EE studies) such as: *An REE study is defined by the analysis of entrepreneurial change processes that alter the inter-relations between the communities of people, their institutional contexts, their organisations and their natural environments that reactively and proactively affect the stability of regional socioeconomic activity.* This stance on the REE reflects the terrestrial view of regional socioeconomic activity but frames it in terms of the dispersion of communities across the globe with different natural environments that may each vary also in institutional form. It also acknowledges that the focal biology in this type of ecosystem is people and what they do within the community to sustain and change the ecosystem as they form and dissolve organisations that support their community. Next, it emphasises that the institutional and natural contexts or settings underpin the locational boundary of the community. Lastly, it notes that the focus is on the stability of the socioeconomic activity that provides the ongoing supporting infrastructure for a community’s social arrangements, highlighting its interdependence with the institutional and natural conditions. From within this definition, we also confront the challenges imposed by partitioning of different interests stemming from the fact that any economy, at its heart, will place various demands on its natural resources and physical environment. It will hold differences in social ambitions, norms, arrangements and expectations for subsequent returns. Each economy will be served differently by, and have different expectations of, knowledge, science and technology. Lastly, and perhaps the agenda that seems to receive the most attention from an entrepreneurship standpoint, is that the economics of the community in terms of wealth production, distribution, and utility will have differential profiles.

#### Check 2: Theoretical zones and boundaries correspondence

Given that studies in FEs and REEs share an interest in spatially bound contexts, we next check whether there is potential correspondence between how the zones and boundaries of a forest are defined with similar extensions to the REE. Two concepts are important here in FE terms. The first being the climatic zone the forest is situated in that provides the conditions for particular forest characteristics, and the second concept is the breadth and range of the species that define the forest area.

In REE terms, we proposed earlier that a climatic zone may share similarity with the industrial arrangements that support business and entrepreneurial activity. In particular, the dominance of an industry profile may present an industrial zone boundary condition for the REE. However, the analogy is problematic. Just as the studies of forests are about how trees support the diversity of organic and inorganic relations, the REE definition suggests that the socioeconomic activity of industry supports the diversity of social and economic relations in regions. In turn, industry is influenced by the natural and institutional contexts or settings like the trees of forests are influenced by climatic zones. In short, if industry is a supporting socioeconomic activity arrangement in a region, it cannot also be the determining zonal characteristic of itself. Therefore, this literal interpreted analogy must be rejected.

In exploring the climatic zone proposition, an REE equivalent can also be considered through natural and institutional contexts. It suggests that the climatic zone equivalent may be either the natural advantages of a place or its institutional formal and informal ‘rules’. The natural conditions for an REE boundary may include conditions such as local climate (advantageous for primary industry, for instance) and/or factors such as deep ports or harbours (relevant to manufacturing, transport and logistics) or other local geographic factors (lakes or fertile valleys) that support a diversity (or in some cases dominance) of human activity and business forms. In these terms, the climatic zone of a FE’s micro-climate and geographic positioning may not be that dissimilar to the considerations necessary for an REE. That is, the natural environment may be foundational to the establishment of a socioeconomic community in some regions or be critical to sustaining economic activity such as may be evidenced in tourism-dependent locations.

The second consideration includes the social, cultural, economic and political context that may influence the boundary conditions for a socioeconomic colony (Spigel, 2017). This implies that an REE may have an extra layer of zonal conditions, being the institutional setting (North, 1990; Scott, 1987) that comprises the political, social and legal, formal and informal ‘rules’ that contribute to the context of new venture creation, opportunity, support and legitimacy, shaping and influencing the mental models of actors (Denzau & North, 1994). Neumeyer et al. (2018), who examined the issue of social boundaries in EEs, concluded that boundaries are subject to groups of like ventures (e.g. high tech, high growth or lifestyle ventures), the common forms of institutional support services (e.g. universities or government agencies) or individual level characteristics (e.g. race, ethnicity, or gender). This resonates soundly with the dispersion of a common tree species in forest ecology as defining a boundary. Therefore, the boundary of an REE may propositionally be also influenced by groups of linked economic actors (firms and transacting organisations), who are in turn influenced by institutional attributes that may be dominant within a community, and these may be influenced furthermore by the natural conditions.

The combination of the natural and institutional settings proposes limits to various socioeconomic activity and therefore suggests there is no direct one to one correspondence between the FE and REE at this level despite the apparent metaphoric similarities. Unlike the FE model developed in this article, the REE has an additional institutional layer on top of the natural contexts that influences the form, type and spread of socioeconomic activity in a regional boundary. The boundary conditions of an REE are in effect set by the range of natural and institutional settings that support the particular community in focus for the study.

#### Check 3: The change dynamics correspondence

With respect to the change mechanisms imposed on the REE, we propose that the FE model equivalence falls short in its explanations of change for a socioeconomic region.

**Fig. 3:**
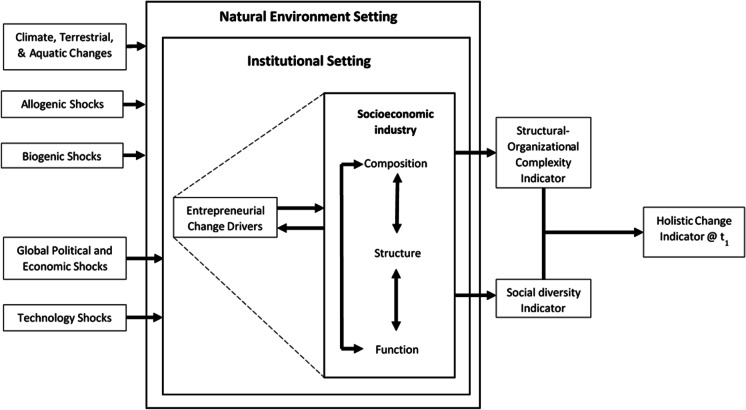
The theorised regional entrepreneurial ecosystem (REE) conceptual model ( Source: authors)

At a global level, like in an FE, an REE could be affected by factors generated externally to the REE boundary that are beyond the control of the actors within a region. For example, global political economies may alter economic conditions (erection of trade barriers or sanctions) or opportunities (free trade agreements) or introduce advances in technology. However, unlike our literal interpretation of the analogous conceptual model above that suggests global power (in terms of political economy) and technology replace the millennial-paced climate change as a factor, a realist perspective will accept that climate change is a highly relevant influence in the REE model. We note that both millennial-paced terrestrial and aquatic change due to shifts in climate will and can dramatically affect the socioeconomic activity in a particular region or area. Therefore, the longer-term changes in an REE include climate and the geopolitical and technological influences. Although we note that geo-political along with technological influences are also potentially more frequent and fall within shorter temporal cycles than centuries or decades, we therefore propose two further origins of more frequently experienced shocks for an REE that may cause or induce change. The first we might call political/economic (produced by external political and economic shifts of which the global financial crisis would be an example) and, the second, technological (produced externally by advances and changes in technology like information technology or artificial intelligence) (see Fig. 3).

In FE terms, we confirm the presence of more frequent shocks (century or decade intervals) as termed either allogenic or biogenic, and each has a distinct form either physical (wild fires and floods) or biological (Covid-19 or insect plagues) that will influence the regional socioeconomic community. Therefore, we retain these aspects in the REE model.

The FE literature also introduces an autogenic change process, and as noted previously, this offers perhaps the most significant analogous transference to the REE. While acknowledging the diversity of views on entrepreneurship, two recent broad reviews suggest something very specific in terms of what entrepreneurship offers an ecosystem. The first, outlined by Audretsch et al., (2015, p. 709) suggests that entrepreneurship is inherently multi-level and involves the ‘static element [that] creates an optimal environment for entrepreneurial action, and the dynamic elements [that] are embodied in the spillover impacts’. The second, by Mayer et al. (2018), suggests ‘the creation of new enterprises or the creation of entrepreneurial activities in existing enterprises … generate indeterminate uncertainty’. Embedded in both these views is the notion that the ‘entrepreneurial’ is not only about the static view to optimise entrepreneurship, but it also includes its influence on change within the bounded study of the EE. In other words, we suggest it is an autogenic change in a similar way to the terms of the FE literature.

When entrepreneurship is viewed as a mechanism of change, the study of an REE should then examine the influence of entrepreneurial activity on the changes in the structure of elements contributing to the socioeconomic community. The role of new ventures and entrepreneurial activity within a socioeconomic ecosystem is to seize new and emerging opportunities either through knowledge spill-over or by responding to experienced or perceived changes and disruptions external to the region. Hence, entrepreneurship creates and/or responds to conditions of uncertainty that alter the dominant socioeconomic activity within the ecosystem boundary. This framing is an alternative to the EE thinking about entrepreneurship comprising an entrepreneurial actor responsible for economic and other ‘value creating’ outcomes. By contrast, an REE framing suggests that ‘the entrepreneurial’ within a regional socioeconomic system is an endogenous change agent responsible for shifts in socioeconomic composition, structure and function that may be observed in many dimensions, in various ways and by many disciplines.

Studies that illustrate this thinking are found in the allied fields of industry agglomerations, clusters and complex systems. When considering local economic structures and agglomeration economies, the creation of innovative start-ups varies in response to the different local development conditions (Capozza et al., 2018). Innovative start-ups have also been instrumental in both creating diversification (through capitalising on opportunities influenced externally to the community boundary) and specialisation (seizing opportunities to build and reinforce strengths found within a community). Similarly, studies of cluster formation (the dynamic and emergent state pre-agglomeration and observable clusters) is seen to be influenced by strong entrepreneurial influences such as start-ups, network building, forming actor linkages and new knowledge that enables branching of local industry and propels exogenously introduced uncertainty (Li, 2018). Transition studies in complex systems have also directed attention toward the internal mechanisms or power and politics among the networks of both human and non-human agents (Kok et al., 2021). These issues raised across disciplines are more likely to come into focus when considering entrepreneurship as a change mechanism in a regional community through the REE framing of analysis as regions move from one socioeconomic order to another.

#### Check 4: Configurational correspondence

Due to limitations of space, we wish to avoid the deep analysis of each of the highlighted configurational elements to instead examine whether each of the type of elements can be sufficiently described or grouped into composition, structure, and function. To explore this further, we can observe the various standpoints on regional-industrial configurations to consider the consistency or otherwise of the types of elements.

The research relating geographies and industry has a long heritage, and two competing primary narratives emerged from the foundations. Some authors argued that concentration of firms in the same business domain (Arrow, 1962; Marshall, 1890; Romer, 1986) is preferable in a region to drive growth. Others argued the opposite to suggest diversity in urban business and industry has greater merit (Jacobs 1969). The former authors represent the MAR theory, which argues firms in close proximity experience spill-over effects that fuel growth in the regional economy (Glaeser et al., 1992). The latter argument, often accredited to Jacobs (1969), presents the case for variety and diversity in industry that inspires innovation and growth (Glaeser et al., 1992). These two arguments have been more recently re-framed into positions of regional related variety within sectors and unrelated variety between sectors (Frenken et al., 2007). Further advances have supported smart specialisation that focuses on the region’s capacity to ‘leverage existing strengths, to identify hidden opportunities and to generate novel platforms upon which regions can build competitive advantage in high value-added activities’ (Balland et al., 2019, p. 1252). However, despite these advances, there remains a paradox in regional innovation policy and industrial policy (Hassink, 2020; Muscio et al., 2015; Oughton et al., 2002) and a puzzle about the extent to which entrepreneurial processes discover or exploit discovered opportunities (Balland et al., 2019) that remains unsolved. The use of ecological studies to re-frame the problem into an REE explicitly brings into focus the study of the entrepreneurial process and how this process is configured to influence the change dynamics in a region. The REE potentially fills the void left by economic geographers in understanding the regional-industrial dynamics.

Many authors have compared and contrasted different systemic-based views of geographies over the last decade or so. Notably, Cao and Shi (2021) conducted a thorough systematic review to conclude—in agreement with Tallman et al. (2004)—that the thread of argument uniting these studies is that there are geographic boundaries and location specific advantages. The variations between the studies lie in the attention given by each of the author(s) to a number of factors, such as focus and locus of action (O’Connor et al., 2018); the environment, actors, key determinants of system performance and the impacts at micro and macro levels (Pilinkiene & Maciulis, 2014); the transactional costs, resource and knowledge capabilities and distinctions in power and control (Pitelis, 2012); or governance, knowledge, industry, actors, resources and benefits (Spigel & Harrison, 2018). Each of the studies though is concerned with the various parts that differentiate a particular framing of a system. None recognise that the ecological framing is concerned with how the parts work in concert to affect the whole. Adopting an REE approach highlights that there are differences between the wholes (the regional community) and leverages the depth of knowledge accumulated in ecological studies to examine the way communities regionally defined differently configure the parts rather than reducing the differences between the systems to the parts themselves.

It is particularly instructive that borrowing from the biological form of ecological studies (FE) draws attention to different understandings of what changes are important (Chazdon et al., 2016) in an ecology. This is equally reflected in change for the industrial economy and socioeconomic systems that sustain and/or evolve the socio-industrial economy within a particular community. While studies of forests can observe the same resources, which resources selected, how they are arranged and accessed and for what purpose they are used is where the variation in emphasis of the studies is found. We argue that these same configurational differences are also defining factors in distinguishing REEs.

Whether our interests are with analysing industrial districts, clusters, innovation systems, the triple helix, innovation ecosystems, business ecosystem, digital business ecosystem or the EE, the principal reason for doing so is to better understand and design interventions that sustain or change the community and business and/or industry circumstances. Changes affect social standards, living conditions and wealth associated with the community of analysis. Changes in socioeconomic conditions within a geographic region are defined by changes in various elements of analysis of the socio-industrial-economic relationships. Here, we suggest that at a regional level, industry is to be interpreted as the industriousness of the socioeconomic community and not necessarily as a particular industry sector. Regions will invariably comprise multiple industry sectors and at times they may be dominated by an industry sector. However, we intend by industry to represent the socioeconomic activity of the community and the various elements that comprise the socioeconomic infrastructure that supports a community.

Similar variations abound in in the studies of FEs with respect to terminology, measurement, constructs, and methods. While this correspondence of diversity in views and opinions suggests a metaphoric similarity, it does not drill down to the specific correspondence of configurational analysis relating to composition, structures, and functions.

In the EE literature we can find a vast variation in what may be considered elements. Some authors describe the entrepreneurial ecosystem as consisting of individuals with entrepreneurial attitudes, abilities and aspirations (Acs et al., 2014), but others consider a broader pool of elements such as people, roles, infrastructure, organisations and events (Regele & Neck, 2012). Stam (2015) describes a broad set of interdependent actors and Spigel (2017) notes more narrowly nascent entrepreneurs but then broadly adds ‘and others’. Mason and Brown (2014) stress the entrepreneurial nature of both actors and organisations and yet others simply note a group of actors (Cohen, 2006) or a community of complex agents (Roundy et al., 2018) as systemic elements. Although some definitions neglect any specification of what may be defined as elements, it would seem actors are implied as those who are influenced as in Audretsch and Bilitski’s (2016) definition and, as being one of the elements that combine in complex ways in Isenberg’s (2010) definition. Presumably, elements include resources such as finance, labour, skills, and advice that are potentially re-allocated (Acs et al., 2014) or recycled (Mason & Harrison, 2006; Spigel, 2020; Spigel & Vinodrai, 2020) through exchanges within the EE.

These definitional stances represent a variety of elements that do not address more specifically the issues of correspondence with composition, function and structure. It is apparent that the extant EE literature has not moved sufficiently to disentangle the relationships or categorisations of elements, as is highlighted by one notable study:

most studies of the context of entrepreneurship have been qualitative case studies that provide rich descriptions of entrepreneurial ecosystem constructs and elements, but do not provide information about how these constructs and elements are related to each other and to entrepreneurial outcomes. (Stam & Van de Ven, 2021, p. 828)

However, Stam and Van de Ven (2021) attempt to address this by studying the relationship between the combination of ten elements and a productive entrepreneurship output measure using statistical analysis, drawing data from twelve regions in the Netherlands. While they do not find or conclude a distinction among the elements that may specifically correspond with composition, structure and function, they do claim the following:

…that the entrepreneurial ecosystem elements are mutually interdependent and co-evolve in a territory. There is strong interdependence in general, and in three clusters of elements in particular. Talent, entrepreneurial culture and support services are strongly correlated, both simultaneously and over time. The same counts for knowledge and leadership (in innovation projects), also reflecting interdependencies in the knowledge economy. We also find strong interdependencies, both simultaneous and over time, between physical infrastructure and demand. (Stam & Van de Ven, 2021, p. 828)

Not only does the Stam and Van de Ven (2021) study reveals the close correspondence with the inter-relatedness and interdependency of elements as we find in the FE literature, but the three sets of clustered elements suggest a correspondence between compositional, functional, and structural elements. For instance, talent (compositional) clusters with entrepreneurial culture (institutional) and support services (suggestive of a functional element). In other words, when talent is organised by an informal institutional cultural setting and supported by services, one may expect an entrepreneurial output. Similarly, when knowledge is part of the composition of the EE and is facilitated by functional leadership, one may expect a productive entrepreneurial output. Lastly, when there are sufficient physical infrastructure (compositional) elements combined with functional demand, it will likely give rise to an entrepreneurial output.

While we suggest a concordance between the REE and FE configurations, at the conceptual level, further research is needed to be more explicit about what makes up each of the configurational elements in an REE context before any degree of confidence can be expressed in this proposed correspondence. Lastly, the definitional stance and realist ontology also exposes the need for case studies that examine the specifics of regions, their configurations and inter-relatedness as well as empirical studies of data collected across regions to draw attention to the parts within any region that deserve closer scrutiny with respect to their interdependency.

#### Check 5: Dynamic indicators correspondence

The fifth and final check seeks to resolve whether there is a rational correspondence between the FE concept of structural complexity and biodiversity with the REE concepts of structural complexity and social diversity. We have suggested that socioeconomic activity within REE communities may be characterised by the particular configuration of socioeconomic compositional, structural, and functional elements. An EE has been described as a complex adaptive system that brings about new order from the bottom up through interactions among multilevel structures, processes and attributes of the system (Roundy et al., 2018). An EE approach to an REE implies it is inescapably multilevel by its very nature. It follows that there is potentially correspondence with the structural complexity found in FE.

In common with the social sciences of organisational and regional studies, entrepreneurship has long since recognised the existence and interaction of multiple levels (Davidsson and Wiklund 2001). Reflecting the extant literature, the three levels often considered in the social sciences of organisational, regional and entrepreneurship studies (see Kozlowski & Klein, 2000; Lichtenstein 2011; Roundy et al., 2018; Sardeshmukh, O’Connor, & Smith, 2020) are:

- The macro, e.g. the contextual place, institutional and social culture of the EE, the policy settings, and other normative and regulative influences
- The meso, e.g. the interacting economic organisations of place including those that provide resources and support to and for individual entrepreneurs
- The micro, e.g. the population of individuals embodied with intentions, behaviours, attitudes etc. (not necessarily aligned with the macro institutional and social, cultural context) including the individual entrepreneurial actors

The macro level aligns well with the institutional characteristics of social, cultural, political and economic reference points. However, it is readily acknowledged that the term ‘macro’ has instability in its meaning when considered across disciplines, whereby macro could mean anything from industries, interfirm networks, regional clusters, strategic groups, economies, alliances, populations, organisations, fields, societies, labour markets, nations and suprasocietal structures depending upon the disciplinary field referenced (Molloy et al., 2011). From this perspective it is quite reasonable to claim that the ‘macro’-level is not simply a unidimensional level but describes a supra-set of interacting contextual ‘rules’.

Roundy et al. (2017) argue that macro level coherence is built upon shared values and goals, common business models and venture similarities, institutional thickness, community logics and trust. It is apparent in this view that what is referred to as macro represents aggregated formal and informal ‘rules’ or institutions. This has resonance with the extracted FE analogy to the institutional setting. The macro perspective seems to support the idea of a set of conditions within which socioeconomic activities take place. We contend that the macro level accounts for the formal and informal setting of the ecosystem.

At the opposite extreme is the micro level of analysis. In complexity sciences, the bottom-up evolution of new order is termed ‘emergence’ (see Kozlowski & Klein, 2000 for a thorough coverage of the topic at the organisational level and Lichtenstein, 2011, for an equally relevant discussion to entrepreneurship). Of importance for this work is the definitional and compositional view of the micro and whether it may reflect the social diversity indicator relevant for an EE.

A clear link between the micro level and diversity is apparent in Roundy et al., (2017, p. 101) who wrote ‘[d]iversity can manifest in the types of ventures in an EE; however, its genesis is at the individual – “micro” – level of the EE’. Sardeshmukh, O’Connor and Smith (2020) argue that at a micro level, both divergence and non-linear complex dynamics are critical attributes that may be represented by diversity in populations. Perhaps more generally, Bhawe and Zahra (2019, p. 443) make it plain that ‘[e]cosystems that support greater heterogeneity of entrepreneurial activities are vibrant, dynamic, and responsive to changes in global business environments. A lack of heterogeneity cannot buffer firms against changes in technology or customer tastes’. Therefore, the social diversity is potentially a critical indicator of the extent of diversity in socioeconomic activity.

This micro perspective deals with the interests of the individual entrepreneurial actor that both drives and responds to change and uncertainty induced in the REE. Socially diverse entrepreneurial actors are those who reactively and proactively drive autogenic change while at the same time keeping socioeconomic activity running. Therefore, we proffer that populations of individuals form communities aggregate to represent a new industry or participate in existing industries and in turn contribute to other industries and communities that co-exist and are co-located in a region. Meanwhile, the entrepreneurial among them create ventures (whether social, commercial or other) that sustain or change the ecosystem’s industrial dynamics. The greater the social diversity of that community, the greater the potential diversity of ventures will be; and in turn the more evident divergence and non-linear complex dynamics will be, and the more vibrant and responsive the change resulting from shifts in technology, consumer tastes, etc. Hence the micro level for an REE seems to represent the social diversity that corresponds with the biodiversity of an FE.

Considering the concepts of macro and micro levels, we suggest a correspondence with the institutional setting at the macro level and the social diversity at the micro level. However, that leaves a question about whether and how the meso level contributes further to our understanding of the REE. It is apparent that the meso level facilitates interactions (Sardeshmukh, O’Connor and Smith, 2020) and intermediary processes (Roundy et al., 2018) that provide a ‘socially situated cognitive bridge’ (Goswami et al., 2018) between the macro and micro levels. Therefore, the meso level would appear to be an essential component of the conceptual model of an REE, facilitating the transactional alignment between the macro institutional setting and the micro diversity of social actors. This suggests that, in an REE context, a critical configuration of compositional, structural and functional elements is needed that accounts for the connectedness between the macro and micro levels and we might call this structural-organisational complexity.

This nested meso level is particularly distinctive when considering the analogous extraction from the FE and increases the complexity of the ecosystem analysis. In essence, the meso level comprises the organisations that form and/or exist to employ and/or engage the micro level social actors in activities that are in some way regulated by the macro level institutional context. These organisations are a species of their own contending for the resources of the ecosystem. Unlike FEs, the individual communities of species in an REE (humans) form organisations that industrialise and impose conscious and willing actions that compete for and consume resources from within and beyond the ecosystem boundary, at scales sometimes well beyond the needs of the REE communities. FE species (non-human) do not create an artificial resource (money) to facilitate exchange. They do not hold aspirations or desires for goods and services nor create organisations to produce goods and services for monetary exchange. The meso level in this way is peculiar to an REE and is not directly found in a FE. Where FEs do account for human habitation, humans are considered as one species within the ecosystem to be treated as part of the balance of the system (e.g. a home for indigenous people). Alternatively, the FE itself is considered as a resource for the organisations that use the forest resources for an industrial purpose (e.g. a timber resource). However, in an REE there exists any number of organisations that co-exist with the natural environment and the communities of human inhabitants. Organisations are part of the ecosystem analysis, providing an economic engine that sits between the social level of individuals and their communities and the setting of institutional contexts that support and in some way regulate them. The meso level includes organisations, whether they be private or public, for profit or not for profit, service providers, intermediaries, manufacturers or miners. Organisations are a species of their own that reside within the REE. They provide structural ‘habitats’ for socioeconomic activity enacted by humans and also behave as an organism, providing complex arrangements that employ and/or otherwise engage the micro level actors, in competing for and/or providing resources for the REE and beyond.

This leads to the following revised conceptual model of the REE (Fig. 3), derived through closer and realist considerations of the definitional level, the boundary and zonal setting, the dynamics of change, the configuration of activities and the dynamic indicators. It advances beyond the simply constructed interpreted analogous view to extend the conceptual arrangement of ideas that may equate to an understanding of what an REE is and how it may be analysed.

## Discussion: what does the REE framing imply?

Previous attempts at theorising the EE concept in various ways have mostly assumed that the entrepreneurial activity is the objective output and value creation is the outcome of such a system (as nicely conceptualised by Stam, 2015). Our analogous examination of FE suggests that there is an alternate view that refocuses the priority of entrepreneurial activity in an EE to focus instead on the holistic concept of the region’s stability and its changing socioeconomic structure. An REE, as theorised here, focuses on the changing conditions of a region of which entrepreneurship is instrumental in propelling and responding to change. We can differentiate this alternate approach by seeking to understand the holistic ecology of an ecosystem, how this ecology is stabilised or disturbed and how it responds. Sustaining the veracity of entrepreneurship is not the focus of an REE model, but the stability and change of the region is prioritised that considers the influence of entrepreneurship. Table 1 summarises our approach, highlighting the key points of a forest ecology (FE) conceptual model, the analogous construction of a regional entrepreneurial ecosystem (REE) model and the propositionally developed theory of the REE derived through correspondence checks. Thereafter, we discuss the implications of each.

**Table 1:** Summary of analogous REE Theorising

Correspondence	FE conceptual model	Direct analogous FE extraction to an REE	Checked theoretical REE propositions
Definitional level	FEs have two definitional stances. First, an objective utilitarian account of some aspect of the FE; and second, a holistic account of the FE with concern for changes and balance within it	EEs are largely defined by an objective utility of entrepreneurial activity. Extracting the REE analogy creates a second definition that accounts for change in socioeconomic activity	An REE is defined by entrepreneurial change processes that alter inter-relations between communities of people, their institutional contexts, their organisations and their natural environments, that reactively and proactively affect the stability of regional socioeconomic activity
Boundary/Zonal level	The boundaries of an FE are confirmed by the spread of a dominant form of tree canopy cover that supports other biological inhabitants. Across the globe, climatic zones determine the presence or absence of any form of fores	The boundaries of and the zonal conditions for an REE are largely theoretically undefined. We draw from the analogy the provisional idea of institutional contexts and industrial zoning as preconditions to boundary setting	Further analysis suggested that unlike the FE, the boundary of an REE is defined by two determinants: 1) the natural setting that supports communities, industry, and commerce; and 2) the institutional ‘rules’ that influence the geographic spread and coverage of socioeconomic activity
Change dynamics level	The FE draws attention to four levels of change conditions: slow millennial (climate) change, allogenic, biogenic and the internally generated and responsive autogenic change mechanisms	Similar change conditions can be drawn as a parallel for REEs, although the slower millennial changes can also be framed as global shifts in political power and technology and the autogenic change is analogously similar to entrepreneurial change	We propose that REEs exhibit more complex change dynamics than an FE, which extend those of a FE. Geo-political power and technology changes are added to the millennial-paced, allogenic and biogenic changes for an REE. The autogenic influence translates to entrepreneurial change. Together, this amounts to six change drivers that affect the socioeconomics of a region
Configurational level	The FE highlights three interconnected and interdependent features: composition, function, and structure	An REE appears to also exhibit interconnected and interdependent features which may be equally considered as composition, function and structure	We propose that REEs exhibit similar characteristics of composition, function and structure although the specification of these relate to the extent they sustain and vary socioeconomic coverage in the region. The definitions of these elements are unresolved and require further research
Dynamic Indicators level	The FE suggests two primary indicators of FE performance, structural complexity and biodiversity that can be monitored for change over time	An REE can also exhibit structural complexity in terms of industrial settings and a diversity in biological form, being the social diversity of people populating a region that can change over time	We propose that an REE has similarly two indicators of ecosystem performance: the micro level of social diversity of the population; and the structural-organisational diversity at the meso level, which provides habitats for socioeconomic activity. Organisations act as organisms in their own right, producing and consuming resources

### Definitional level

From the stance of an REE, it is notable that the entrepreneurial activity is within the ecosystem rather than an object separable from the ecosystem. Entrepreneurial activity is an endogenous change agent that both responds to shifts within the ecosystem and the external shocks imposed upon the ecosystem; it drives and responds to change in the configuration of compositional, structural and functional elements; organisational-structural complexity; and social diversity from within. Entrepreneurial activity is a balancing mechanism within the ecosystem, influencing community health, wealth and viability by influencing and responding to shifts in compositional elements, the structural arrangements of elements and the functionality of elements. It may also act as a progressive influence upgrading wealth and living standards or retard the advance of socioeconomic development. From this viewpoint, an REE study is concerned with the changes entrepreneurial activity produces, how they occur and what they mean for the ecosystem’s stability and its development, or equally, potential destruction.

The primary implication is that trying to understand regionally based ecosystems through positivist interests in empirical data, and objective partitioned aspects of an ecosystem drawn from EEs, is an incomplete appreciation of the work of forest ecologists and perhaps ecologists more generally. The work of FE studies splits into two camps: those that see partitioned interests of objective priorities imposed upon an FE and those that hold a holistic view of the forest ecology stability. The latter are concerned with species survival or demise and how the changes in ecological stability threaten the survival of some species but advantages others. The balance of the system is in focus and this can only be studied through close inspection of the configurations in the particular forest cases. We contend that this same split in perspectives is true of EE studies, that the REE is a re-framing toward a second holistic understanding of balance within socioeconomic regions and entrepreneurship is the functional element that proactively and reactively changes the socioeconomic system from within.

In effect, we to some extent answer the question raised by Wurth et al. (2021), whether ‘the (current) entrepreneurial ecosystem concept is capable of explaining entrepreneurial dynamics in a variety of contexts or whether it is limited to a small number of regions in high-income countries?’ Our research suggests that the extant EE framing cannot completely explain the dynamics in a variety of contexts because of its objective value framing. In contrast, REE offers a re-framing toward the question of how entrepreneurship influences regional stability and vice versa. It is a step toward re-framing our understanding, and we can learn from ecological studies to explain entrepreneurial dynamics in a variety of contexts. This also necessitates a shift in ontological framing to appreciate the objective and subjective experiences within an REE and seek case studies that adopt a scientific realist approach (Johannessen and Olaisen, 2005). The answer to the second part of Wurth et al. (2021) question on the limits of EE research being only relevant to high-income countries follows from this position. If entrepreneurial ecosystems are appreciated as uniform in theoretical framing but unique in specific configuration, then entrepreneurial ecosystem studies can be applied to all socioeconomic regional ecosystems. By contrast, if a uniform theoretical framing is applied that assumes that there is only one effective configuration to optimise value objectives (e.g. entrepreneurial ventures, economic value or other), then the EE concept may only apply to a limited set of contexts particular to that optimised objective value. Just like rainforests are dependent on particular conditions that optimise structural complexity and biodiversity and are not representative of all forests, we suggest the need to follow the forest ecology work to frame the study of ecologies of entrepreneurship to identify the variation in conditions that support entrepreneurship ecologies and the instrumental factors in changing the ecology.

### Boundary/zonal level

The boundary definitions in ecological works are a frightfully difficult topic to unpack. We propose that, unlike the FE, the boundary of an REE is defined by two determinants: (1) the natural setting that supports communities, industry and commerce and (2) the geographic reach of the institutional ‘rules’. A change in one or the other can signal a shift in ecosystem form and habitat for business and commerce and a potential demarcation of boundary conditions. However, this raises a number of issues in approaching the research of any REE.

To resolve boundary issues and what is counted inside or outside of the ecosystem in the FE works, it is the dominant distribution of tree coverage that matters. Notably, the types and distribution of trees can and do vary. There may be dominant species evenly distributed across a forest and equally there may be small and isolated stands of trees scattered throughout the area defined by the total tree canopy cover. In an REE analysis, we have drawn the parallel of canopy cover to be the coverage of socioeconomic activity to define the spatial area of a region. Clearly, when defined in this way, this can vary massively between small, remote outposts like a rest stop between long distant travel destinations, right through to a thriving metropolis like London, New York or Shanghai or even nation states where the socioeconomic activity and industrial spread can be expansive.

In approaching an ecosystem boundary discussion, it is critical to identify the unit of analysis at a community level and identify who it is that holds that unit of analysis as a priority interest. In many of the EE works, the priority is placed on generating entrepreneurial activity. In the REE framework, derived here, the priority is the stability of a regional socioeconomic community and how entrepreneurship influences that stability. Notably, regions defined by spatial coverage of socioeconomic activity may breach geo-political borders, much as forests pay no attention to state lines (e.g. the Amazon rainforest spans nine countries). Therefore, the boundaries may be driven by any number of priorities such as government interests, organisational interests, specific industry sectors, natural environment agendas and even research interests. Each priority interest may alter the definition of boundary for the unit of specific analysis. This is an inescapable complication of ecosystem research and represents the complications of ecosystems having various parts and various wholes depending upon any particular viewpoint. This is consistent with the realist framing of the REE. The implication is that there is no single answer or correct definition of boundary. However, with each specific community definition, the boundary must be clear, appropriate, and the method of boundary definition, consistent for the research intent.

While in this paper we have articulated an approach to EE analysis that emulates FE studies to yield a conceptual model for an REE, further research of a similar nature could extract methods and approaches to research from other ecological studies. For instance, studying the ecologies that support a specific migratory or nomadic species (Agrawal et al., 2019) might be a better analogy for how we understand the boundary conditions of platform businesses with a community that stretches across regional areas (Autio et al., 2018). Considering exemplar studies of specific stands of trees within a forest (McElhinny et al., 2005) may yield insights into the way we consider the differences in socioeconomic ecologies between neighbouring communities in a single geo-political boundary or those at different levels within the geo-political boundary (Spigel, Kitagawa & Mason, 2020). Eventually, the increasing studies of various ecologies among various communities when combined will start to reveal the bigger picture, coming together like a patchwork quilt to reveal ever larger patterns.

### Change dynamics level

While the high-level definitional principles of the FE and EE may be considered as metaphorically similar, through this study we note that the predominant academic interest, to date, stems from an economic functional and objective perspective of entrepreneurial activity. Our study brings to the surface, not only an opportunity, but it also highlights the importance of developing an REE view that enables a deeper appreciation of the change mechanisms within a regional level socioeconomic ecosystem. The management or governance issues pertaining to stimulating more entrepreneurial activity can be better considered from the vantage point of a particular unitary community. Our conceptual model is one through which the calls for research to examine the integration of entrepreneurial activity governance that takes into account the broader context (Colombo et al., 2019) can be conducted.

Entrepreneurship has been identified as the underlying force driving creative destruction (Schumpeter, 1976), and we argue this is an important function that the ‘entrepreneurial’ serves in an REE context. We must also acknowledge that various studies have offered different views on the function of entrepreneurship in an economy, and some may argue that the lineage of von Mises and Kirzner, for instance, contrasts the disruptive behaviour of entrepreneurs to one of bringing an economic system into balance or equilibrium. However, we can cite Kirzner directly here: ‘… in spite of the contrast with Schumpeter that I emphasised in 1973, the truth is that my understanding of the dynamic market process certainly can (and should!) also encompass the consequences of Schumpeterian entrepreneurship’ (Kirzner, 2009, p. 148). In other words, regardless of the direction of change, whether creating or disrupting equilibrium, entrepreneurs and the entrepreneurial activity within a region provides the dynamics of a market economy inducing stability back from disruption or moving disruption toward instability. A critical point to note is that whether we consider the Schumpeterian or Kirznerian views, entrepreneurs are invariably likely to impact the economic stability of a region. However, economics is not the only factor that determines the stability of socioeconomic activity in a region and markets often can also extend well beyond a regional definition of socioeconomic activity. The REE seeks to explain entrepreneurial change in the specified regional community and takes more than market economics into account.

### Configurational and dynamic indicator levels

Adopting an REE approach suggests that we can sense the impact and efficacy of these dynamics through changes in the structural-organisational complexity and social diversity introduced by configurational differences of composition, structure and function brought about by entrepreneurial activity and external shocks. Further, the structural-organisational complexity is seen at the meso level, while the micro level hosts the social diversity of population. Importantly, the complexity of the meso level of organisations will influence the REE performance through alignments and misalignments between the micro level of population and individual activities and the macro formal and informal institutions that create the ‘rules’ for those activities within the region. The difference of the compounded variation of structural-organisational complexity and social diversity between time periods may be an important indicator of regional entrepreneurial change. From this viewpoint, the measure of performance of an REE is not the amount of entrepreneurial activity or the value created (or destroyed) alone but the progressive, stable or possibly regressive change in structural-organisational complexity and social diversity. To explore this further, ecological studies of population change such as that by Berryman (1999) or Briscoe et al. (2021) may be extremely useful to extract methods, ideas and clues about indicators, empirical methods and theories that can be related back to industries, firm populations and changes in populations of specific actors or entrepreneurs.

### Operationalising the model

The REE provides an alternative approach to other framings for EE research. We do not propose that it diminishes the value of other approaches, but we do propose that it is different and necessary as an additional tool in the tool kit to understand entrepreneurial ecologies. It highlights change and the dynamics of socioeconomic systems. While other EE approaches for instance focus on ‘productive entrepreneurship’ (Stam, 2015; Wurth et al., 2021) as the central concept and value creation as the *raison d’etre*, the REE acknowledges that the stability of socioeconomic systems is of equal concern. An example of how focusing on productive entrepreneurship can be harmful to a socioeconomic ecosystem is where fish stocks are depleted and fishing communities decline through productive entrepreneurship that increases the scale of fishing or new technologies to catch or find schools of fish or just too many productive entrepreneurial ventures that overpopulate one area. The change in ecosystem (ΔEcosystem) represents an analysis of the stability and instability of the socioeconomic structure itself. The research of an REE explains the trajectories and implications of prioritising one or another value objective in terms of the effect of the entrepreneurial behaviours on the socioeconomic community. An REE framing completes the objective views and makes them relevant to the ecology of human and place interaction.

To provide guidance for the future development of the model, we offer the following as an initial means to operationalise the model in formulaic terms. There is no doubt further work and research and development of the model are required. However, in summary, the following may be useful as a theoretical derivation for examining REEs.

$$\begin{array}{c}\Delta Ecosystem\approx f\Delta \left(\mathrm{Structural Organisational Complexity }.\mathrm{ Social Diversity}\right)\mathrm{t}1-\mathrm{t}0\\ =f\Delta \left(\frac{\mathrm{Socioeconomic industry }\left\{\mathrm{Composition},\mathrm{Structure},\mathrm{Function}\right\}}{\mathrm{Entrepreneurial Change }\left\{\mathrm{Composition},\mathrm{Structure},\mathrm{Function}\right\}}\right)t1-t0\\ \begin{array}{c}\times Entrepreneurial change \{Composition, Structure, Function)\times \Sigma Ext Change\\ \times Institutional Settings \times Natural Settings\end{array}\end{array}$$

To define our terms:

- Ecosystem is the regional socioeconomic community defined by the boundary of interest as the unit of analysis limited by the socioeconomic industry spread supported by natural and institutional settings and subject to external change.
- Natural settings refer to elements of geography, micro-climates, physical landscape features or similar that support and influence the existence of a socioeconomic community.
- The institutional settings are the formal and informal rules that regulate behaviour either through normative or legal means shared and characteristic of the socioeconomic community.
- The external change includes the change influences on the socioeconomic community beyond the control of the community and introduced into the boundary conditions by such things as climate change, allogenic and biogenic events, technology shifts or political power and trade influences.
- Structural organisational complexity is the mix of organisations that engage humans in industrial activity and serve as the mechanisms through which institutional, productive and change activities are coordinated and enacted.
- Social diversity is the mix of human representation that input into the regionally defined socioeconomic industry and entrepreneurial change.
- Socioeconomic industry is the productive and routine activity that normally sustains the community within the regional definition comprised of a configurational set of composition, structure and function.
- External change is the sum of the external pressures on the defined regional community to change.
- Entrepreneurial change is the internal pressures imparted by the entrepreneurial activities across various levels that respond to external change pressures or initiate and drive internally derived change comprised of a configurational set of composition, structure and function.
- Composition comprises the human and non-human physical components identified in the regional community before incorporated into structure or endowed with function.
- Structure is the organisational arrangements of compositional elements that combine and coordinate through a socioeconomic entity or programmatic form to perform or deliver some function(s) within the regional community definition.
- Function is the actual intended or unintended impact of actions imparted by either operating structures or potentially single compositional elements that deliver or contribute to the productive or change dynamics of the socioeconomic ecosystem. We note that there are two primary functions, the productive routine socioeconomic function and the entrepreneurial change function and sub-functions will be apparent in systemic combination to deliver the two primary functions.

While this summary presentation is conveyed in formulaic terms, the analysis is actually systemic and informed by the objective and subjective realist positions of those within the regionally defined community. The conceptual model provides an overarching set of ideas that potentially represents a consistent form of analysis for the entrepreneurial changes in regionally defined socioeconomic industry that we call the REE. However, just like forest ecologists need to engage with the particular ecosystems, habitats and populations of their interest, the entrepreneurship ecologist must engage with the particular REE in focus for the study to understand the complex associations that are represented in this simplistic representation.

The programmatic re-framing of our research offers a set of theoretical ideas and concepts that frame the analysis of REEs applicable generally to regional contexts through which the specific and idiosyncratic relationships and entrepreneurial change dynamics of communities can be uncovered. However, it is not intended to offer a universal model that maximises or optimises the value created by entrepreneurship in all EEs or to generalise strict patterns of relationships that drive and deliver entrepreneurship within all EEs. Each REE will vary across the same categories of ideas to form unique configurations of relationships and entrepreneurial change. The work of the entrepreneurship ecologist using this framework is to diagnose and predict how entrepreneurship will and can be developed through experienced and imposed change pressures in a specific regional community. Thereafter, the value effect of entrepreneurship can be assessed with respect to change in the ecosystem and socioeconomic industry and consequently the community’s fortunes and trajectories with it.

## Conclusion

We have introduced in this article a view of an EE that was inspired by a popular rainforest metaphor. The theorising process undertaken suggests that in understanding and explaining an ecosystem, a realist ontology that accounts for both the objective and subjective views are necessary to comprehend the parts and the whole as it relates to regional socioeconomic activity. REE research, therefore, inspires a multi-paradigmatic research agenda (Gioia & Pitre, 1990; Gioia, 1999) and consequently should aim toward building a scientific realist view (Johannessen & Olaisen, 2005) to account for specific REEs.

The contribution of this article is the introduction of a theoretical framing that adopts a regionally defined community stance. While we acknowledge our limitations of the breadth and depth of coverage of the forest ecology body of knowledge, we believe we have managed to extract the essence of a FE sufficiently to ground an analogous theoretical stance. The approach is intended to leverage a foundational counterpoint to current studies and is not intended to incorporate a full treatise of forest ecology. The proposal provides a unitary community socioeconomic ecosystem concept that we refer to as the REE. The analogous theorising borrows and learns from the field of ecological studies. Consequently, we expose the variety of approaches to understanding ecologies that embraces flexible approaches to boundaries and methods in order to appreciate the complex dynamics between organic and inorganic relations. Through the interpreted analogy and subsequent realist theoretical analysis, we derive a propositional ecological form, the REE conceptual model, as an alternate framing to the dominant EE objective value approach to an entrepreneurial ecology analysis.

From this point we invite researchers to, first, further test, validate, refute and/or develop the REE model and, second, explore other ecological framings to expand our appreciation of the complex interlocking systems of socioeconomic activity. While economic geographers have struggled to resolve the paradox between regional investments and regional change and development, broadly, the EE approach offers a potential alternative by offering a more inclusive means of appreciating the human and regional interactions. However, the extant EE literature has tended to frame the analysis through the lens of increasing entrepreneurial activity to achieve gains in objective economic and/or other value measures. While each objective value priority has merit, by approaching the topic without an appreciation of the balance between the human subjectivity of social, economic, technological, and natural environment, we are at risk of missing the deeper and more complex connections between the entrepreneurial inputs and the evolutions of regional socioeconomic systems. If we wish to adopt an ecosystems view of entrepreneurship, then we suggest we need a robust engagement with the literature of ecologies to learn from and adapt our approaches and methods. We hope our modest effort inspires others to explore and learn more about ecological approaches to improve our abilities to offer insight into the entrepreneurial and evolutionary impacts imposed upon variously defined socioeconomic communities.
